# Green synthesis of a dual-functional sulfur nanofertilizer to promote growth and enhance salt stress resilience in faba bean

**DOI:** 10.1186/s12870-024-05270-7

**Published:** 2024-06-26

**Authors:** Asmaa M. Khalifa, Fatmah A. Safhi, Doaa E. Elsherif

**Affiliations:** 1 Botany and Microbiology Department, Faculty of Science, Al Azhar University (Girls Branch), Cairo, Egypt; 2 Department of Biology, College of Science, Princess Nourah bint Abdulrahman University, P.O. Box 84428, Riyadh, 11671 Saudi Arabia; 3 Botany Department, Faculty of Science, Tanta University, Tanta, 31527 Egypt

**Keywords:** Salt stress, Green synthesis, Sulfur nanofertilizer, Antioxidant enzymes, Gene expression, *Vicia faba* L

## Abstract

**Background:**

Salinity is a major abiotic stress, and the use of saline water in the agricultural sector will incur greater demand under the current and future climate changing scenarios. The objective of this study was to develop a dual-functional nanofertilizer capable of releasing a micronutrient that nourishes plant growth while enhancing salt stress resilience in faba bean (*Vicia faba* L.).

**Results:**

*Moringa oleifera* leaf extract was used to synthesize sulfur nanoparticles (SNPs), which were applied as a foliar spray at different concentrations (0, 25, 50, and 100 mg/l) to mitigate the negative effects of salt stress (150 mM NaCl) on faba bean plants. The SNPs were characterized and found to be spherical in shape with an average size of 10.98 ± 2.91 nm. The results showed that salt stress had detrimental effects on the growth and photosynthetic performance (Fv/Fm) of faba bean compared with control, while foliar spraying with SNPs improved these parameters under salinity stress. SNPs application also increased the levels of osmolytes (soluble sugars, amino acids, proline, and glycine betaine) and nonenzymatic antioxidants, while reducing the levels of oxidative stress biomarkers (MDA and H_2_O_2_). Moreover, SNPs treatment under salinity stress stimulated the activity of antioxidant enzymes (ascorbate peroxidase (APX), and peroxidase (POD), polyphenol oxidase (PPO)) and upregulated the expression of stress-responsive genes: *chlorophyll a-b binding protein of LHCII type 1-like (Lhcb1), ribulose bisphosphate carboxylase large chain-like (RbcL), cell wall invertase I (CWINV1), ornithine aminotransferase (OAT),* and *ethylene-responsive transcription factor 1 (ERF1)*, with the greatest upregulation observed at 50 mg/l SNPs.

**Conclusion:**

Overall, foliar application of sulfur nanofertilizers in agriculture could improve productivity while minimizing the deleterious effects of salt stress on plants. Therefore, this study provides a strong foundation for future research focused on evaluating the replacement of conventional sulfur-containing fertilizers with their nanoforms to reduce the harmful effects of salinity stress and enhance the productivity of faba beans.

## Background

Climate change poses a significant challenge to global agriculture, and salinity stress is emerging as a major threat to crop productivity in this changing climate [1]. According to the Food and Agricultural Organization (FAO), a significant portion of the Earth's land area is affected by salinity stress. Over 424 million hectares of topsoil and 833 million hectares of subsoil, covering 85% of the global land area, are affected by salinity stress [2]. This widespread issue primarily arises from irrigation practices that utilize water with high salt content and the intrusion of saline water from the sea and rivers. Based on projections, the extent of arable land affected by salinization is expected to surpass 50% globally by the year 2050 [3].

Salinity is indeed a significant obstacle that negatively impacts the physiological and biochemical characteristics of plants, leading to a decrease in yield [4]. The presence of high levels of salts in the soil causes an excessive amount of Na^+^ in the cell, which can disrupt the homeostasis of other essential ions such as potassium (K^+^) and calcium (Ca^2+^) within plant cells [5]. This imbalance interferes with various cellular functions and can impair the overall growth and development of plants. Furthermore, salinity leads to an increase in the production of reactive oxygen species (ROS), which, in turn, negatively impacts plant growth [6]. The excessive production of reactive oxygen species (ROS) in plants is influenced by factors such as osmotic pressure and ionic toxicity, resulting in oxidative damage [7]. These ROS can cause harm to plant tissues, disrupt the structure of double-helical DNA, interfere with the integrity of the phospholipid bilayer and degrade proteins [8].

Plants principally respond to salinity induced oxidative stress via various defensive mechanisms comprising different enzymatic (APX, POD, PPO, etc.) and nonenzymatic (ascorbic acid, phenolic acids, flavonoids, alkaloids, etc.) antioxidants [9]. This response involves changes in various morphological and/or biochemical processes according to the plant species, type of stress, and exposure time to the stress [9, 10]. For instance, photosynthesis, the basis of plant growth, is one of the most stress-sensitive biochemical processes in plants [11]. Salt stress resilience is controlled by stress-responsive genes and regulatory pathways [12]. This acclimation is commonly mediated through the accumulation of osmolytes such as sugars and amino acids, especially proline [13]. However, these natural defense mechanisms are often insufficient to counteract the deleterious effects of high salt concentrations in the soil. Consequently, there is a pressing need for innovative approaches to improve plant tolerance to salt stress and sustain agricultural productivity in saline environments.

In agriculture, nanotechnology is extensively applied in the form of nanofertilizers and nanopesticides. These nanoforms can monitor nutrient levels, stimulate growth and productivity, and enhance plant stress resilience [14, 15]. Nanofertilizers have unique physicochemical properties, such as a high surface area and high reactivity, which enable targeted delivery of nutrients and bioactive compounds to plants [16]. Moreover, nanofertilizers have the potential to reduce the impact of oxidative stress and enhance biochemical activity, such as increasing the levels of proline and chlorophyll [17]. They also help maintain the relative water content, regulate salt toxicity, and reduce the accumulation of harmful substances such as melondialdehyde and hydrogen peroxide [18]. Additionally, nanofertilizers play a crucial role in maintaining ionic equilibrium in plants when they are subjected to stressful conditions [17]. As a result, the use of nanofertilizers for enhancing stress resistance in plants has garnered significant attention in contemporary times.

The green synthesis of nanoparticles (NPs) with a size less than 100 nm is a promising biotechnological research trend [19]. This biofabrication method is eco-friendly, nontoxic or less toxic, effective, and cost-effective compared to other conventional physical and chemical methods [20, 21]. Currently, the plant-based green synthesis of nanofertilizers, such as sulfur, is receiving increasing attention due to its simplicity, cost-effectiveness, reliability, and eco-friendly nature [22]. The active constituents of plant extracts play a key role in the stabilization of SNPs [23]. *M. oleifera* leaves contain various bioactive compounds that have reducing and stabilizing effects during the synthesis of nanoparticles [24]. Sulfur (S) plays a vital role in the growth and development of plants because it is a fundamental component of proteins, amino acids, and various secondary metabolites [25]. Sulfur has a vital and essential role in several plant processes like cellular structure, electron transport, and metabolic pathways [26]. Recent reports have shown that S supplementation enhances the photosynthetic rate in plants by improving nitrogen assimilation and chlorophyll biosynthesis [27].

Faba bean (*Vicia faba* L.) is widely cultivated as the fourth most vital cool-season legume crop, grown globally under diverse cropping conditions and environments [28]. This plant is widely used for human and animal feed in many countries because of its high contents of organic materials (e.g., proteins, essential amino acids, thiamin, folic acid, and tocopherol) and minerals (e.g., iron and zinc) [29, 30]. The performance of this legume crop in agroecosystems is markedly affected by the surrounding environmental conditions. To meet global food and freshwater demands and achieve sustainability and food security, researchers are striving to mitigate the deleterious effects of saline water stress on the productivity of such important crops as faba bean [27, 31]. In light of these knowledge gaps, this research aimed to investigate the efficacy of green sulfur nanoparticles (SNPs) as ecofriendly nanofertilizers for enhancing salt stress tolerance in faba bean and to elucidate the underlying physiological and molecular mechanisms involved. By addressing these research questions, this study seeks to contribute to the development of sustainable agricultural practices that can mitigate the adverse effects of soil salinity and ensure global food security in the face of climate change.

## Materials and methods

### Green synthesis of sulfur nanoparticles (SNPs)

The fresh leaves of *M. oleifera* were washed many times with distilled water. To make the leaf extract, 5 g of ground leaves of *M. oleifera* were boiled in 100 ml of distilled water at 60 °C cooled to room temperature, and centrifuged at 4000 rpm for 15 min.

**Fig. 1:**
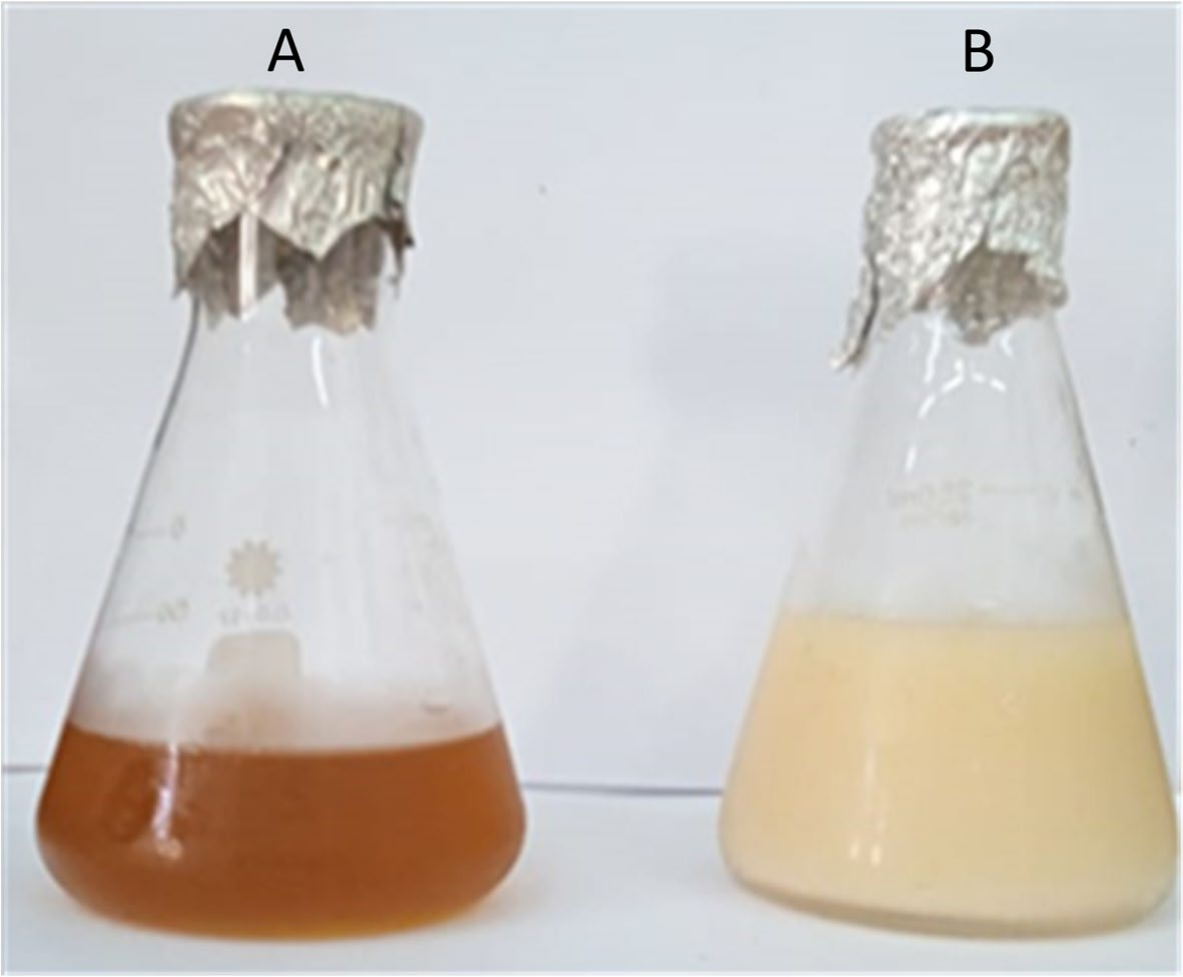
Changes in the color of *M. oleifera* leaf extracts from (**A**) brown to (**B**) yellowish-white

The synthesis of SNPs was dependent on the disproportionation reaction of sodium thiosulfate pentahydrate (Na_2_S_2_O_3_.5H_2_O) in an acidic medium to sulfur and sulfonic acid [32, 33]. Sodium thiosulfate pentahydrate (5 g) was dissolved in 100 ml of *M. oleifera* leaf aqueous extract with moderate stirring for 15 min at room temperature. The precipitation of sulfur particles was performed by the dropwise addition of 10% HCl with stirring until a color change was observed (Fig. 1). The precipitate was collected by centrifugation at 10,000 rpm for 15 min, repeatedly washed with deionized water, and finally washed with absolute ethanol. After purification, The SNPs were heat-dried at 60 °C for 5 h, to produce a white-yellow powder of sulfur nanoparticles which was subsequently characterized.

### SNP characterization

The green synthesized of SNPs was confirmed by a UV‒-visible spectrophotometer (Shimadzu 240). Then X-ray diffractometers (GNR-APD 2000 pro,) were used. The morphology and size of the SNPs were confirmed by transmission electron microscopy (TEM). The material suspensions were sonicated for 20 min on an ultrasonicator (Crest Ultrasonics Corp., New Jersey, USA). Then, afew drops were added to a carbon-coated copper grid and left to dry. Then the grid loaded with the sample was studied by HR-TEM (JEOL, JEM-2100, Tokyo, Japan), operated at 200 kV. An FT-IR spectrophotometer (Bruker Tenor 27) was used to determine the chemical composition of the nanopowder. The resulting FT-IR spectra characterize the molecular structures and chemical bonds of the synthesized nanopowder and leaf extract of *M. oleifera*.

### Plant materials and experimental design

Faba bean seeds (*Vicia faba* L. cv. Giza-716) were obtained from Gemmeiza Agriculture Research Station, El-Garbia, Egypt. The seeds were sterilized for 15 min in 5.4% sodium hypochlorite (NaOCl), followed by five sterile distilled water washes. Then, 3–4 sterilized seeds were sown per cultivation pot (15 cm depth and 20 cm diameter) which contained 5 kg of air-dried soil (sand: clay, 3:1 v/v, pH 6.78). The seeds were irrigated regularly with tap water for 2 weeks. After this period, the seedlings were subjected to two salt concentration treatments: control (0 mM NaCl) and 150 mM NaCl, with 70% of their field capacity (FC). The NaCl concentration was gradually increased by adding 50, 100, and finally 150 mM NaCl to the pots on alternate days over 10 days to reach the desired 150 mM NaCl concentration, thereby avoiding osmotic shock. After that, 150 mM NaCl-irrigated pots were subjected to foliar spraying of SNPs twice weekly in 4 groups as follows: 150 mM NaCl, 150 mM NaCl + 25 mg/l SNPs, 150 mM NaCl + 50 mg/l SNPs, and 150 mM NaCl + 100 mg/l SNPs (The salinity stressed plants were irrigated with tap water or 150 mM NaCl alternately every week until harvest). After 60 days of sowing, the plants were harvested to estimate the following parameters:

### Growth parameters

The harvested plants were thoroughly washed with water. The shoot length (cm) and root length (cm) were measured. Additionally, the fresh weight (g) and dry weight (g) of the entire plant were measured to assess its growth performance.

### Measurement of photosynthetic performance (Fv/fm)

The photosystem II (PSII), an indicator of photosynthetic performance, was evaluated in the fourth dark-adapted leaf by a digital fluorometer (OS-30 P, Hudson, USA). (FV); Minimum value for chlorophyll fluorescence and (Fm) maximal possible fluorescence value.

### Osmolyte contents

In this study, the total soluble sugar content was measured via the phenol–sulfuric acid method [34]**.** The total soluble sugar content was determined at 490 nm in a spectrophotometer (UVISCO model V-1200) and is expressed as mg/g d.wt.

The amino acids content of the ethanolic extract was measured as described Lee and Takahashi [35] using a spectrophotometer. The amino acid content absorbance was content absorbance was observed at 520 nm and is represented as mg/g d.wt.

The proline content in faba bean leaves was determined using described by Bates [36]. Dry faba bean leaves were extracted by grinding in a 3% sulfosalicylic acid watery solution. Then, the leaf extract was boiled in an acid ninhydrin reagent for 1 h. The obtained chromatophore was extracted with toluene, and measured at 520 nm, which was computed as mg/g d.wt.

Glycine betaine (GB) in the watery extract of the dry plant was detected using the method described by Grieve and Grattan [37]. The extracted samples were diluted in a 1:1 ratio with 2 N HCl and combined with cold KI-I2 reagent while being continuously stirred. The resulting crystal was redissolved in 1,2 dichloroethane and then estimated at 365 nm, and the result was calculated as mg/g d.wt.

### Oxidative stress biomarkers

The hydrogen peroxide (H_2_O_2_) content in fresh leaves of faba bean was determined as described by Junglee et al. [38]. Leaves (200 mg) were groun with 1 mL of extraction solution comprising 0.1% (w/v) trichloroacetic acid (TCA), 1 M KI, and 10 mM potassium phosphate buffer (pH 5.8) for 10 min. The extract was estimated to contain H_2_O_2_ at 390 nm.

The content of MDA was ascertained by the reaction of thiobarbituric acid (TBA), as determined by Heath and Packer [39]. Leaves were extracted in a 5% (w/v) TCA solution. The reaction mixture consisted of the sample extract and 0.67% (w/v) TBA, which was then boiled for 20 min and cooled. The sample absorbance was measured at two wavelengths, 532 and 600 nm, and then calculated as n mol/g.f.wt.

### Estimation of nonenzymatic antioxidant compounds

The phosphomolybdenum method was used for total antioxidant capacity (TAC) determination. TAC reagent was prepared by combining ammonium molybdate tetrahydrate (4 mM), sulfuric acid (0.6 M), and sodium phosphate dibasic solution (28 mM) at a ratio of 1:1:1 [40]. Approximately 100 μl of methanolic extract was added to TAC reagent (1 ml) and boiled for 90 min. The TAC was measured at 765 nm.

Ascorbic acid was evaluated according to Oser [41]. Leaf tissues were extracted in 5% sulfosalicylic acid. The reaction mixture consisted of 2% Na- molybdenate, 0.15 N H_2_SO_4_, 1.5 mM Na_2_HPO_4_, and leaf extract. The reaction mixture was heated at 60 °C in a water bath for 45 min, cooled and centrifuged. The change in optical density was monitored at 290 nm. The ascorbic acid content was calculated as mg/g f.wt.

### Extraction and assay of antioxidant enzymes

The enzyme extracts were prepared by grinding 0.5g of fresh leaf tissue with 8 ml of 0.1 M K-phosphate buffer (pH 7). The fresh leaf extract was then centrifuged at 10,000 rpm at 4 °C for 30 min, and the resulting supernatant was subjected to three enzyme assays. Enzyme activity was calculated as μM/g.f.wt min^−1^.

Ascorbate peroxidase activity was determined as described by Nakano & Asada [42]. The reaction mixture comprised 50 mM sodium phosphate buffer (pH 7.0), 0.1 mM H_2_O_2_, 0.1 mM EDTA, 0.5 mM ascorbic acid, and 0.1 mL of plant extract. The change in optical density was monitored at 290 nm.

The activity of peroxidase was estimated according to Kato and Shimizu [43]. In brief, the reaction mixture (11.8 mM H_2_O_2_, 7.2 mM guaiacol, and 100 mM K-phosphate buffer, pH 5.8) to 0.1 ml of enzyme extract. The POD activity was mixed with at 470 nm.

The activity of polyphenol oxidase was measured by the method of Kumar and Khan [44]. The PPO assay was performed by mixing the enzyme extract with the reaction mixture (0.1 M K-phosphate buffer, pH 6, and 2 mM pyrogallol). The reaction was terminated by the addition of sulfuric acid (2.5 N), and the absorbance at 420 nm was determined.

### Extraction of total RNA, cDNA synthesis and RT-PCR:

The extraction of RNA from faba bean leaves was carried out with an RNase Mini Kit (Qiagen). To obtain complementary DNA (cDNA) reverse RNA transcription was performed in a thermocycler (MJ Research, Inc., PTC-100™ Programmable Thermal Controller, USA). The primers used are listed in Table 1.The qRT-PCR were performed in three replicates by using SYBR Green PCR Master Mix (Fermentas, USA). A Rotor-Gene 6000 (QIAGEN, ABI System, USA) was used for the reaction. The gene GAPDH was used as a reference gene**.** The relative expression and calculatin content of the studied genes were quantified as described by Livak and Schmittgen [45].

**Table 1:** Specific sequences of primers used in this study

Gene name	Abbreviation	Forward (F) and reverse (R) primer 5′–3′
Reference gene	***GAPDH***	F: 5′-AAGGTTATCAACGACAGGTTTG-3′ R: 5′-ATACCCTTAAGCTTGCCTTCTG-3′
Chlorophyll a-b binding protein of LHCII type 1-like (LOC114182484)	***Lhcb1***	F:5′-GGCTTTTGCTGAGTTGAAGG-3′ R: 5′-GTAAGCCCAGGCATTGTTGT-3′
Ribulose bisphosphate carboxylase large chain-like (X01167)	***RbcL***	F:5′-CTTGGTACCATCCAACCAATTCA-3′ R:5′-GCTTGGAACCCAACCTTTGC-3′
Cell wall invertase I (Z35162)	***CWINV1***	F: 5′- GGGTTGGACCGTTTGGACTT -3′ R: 5′- CACGCCCGATTAAAACCATACT -3′
Ornithine aminotransferase (loc107482012)	***OAT***	F: 5′-GAATACTGGCGCTGAAGGTGTG-3′ R: 5′-AGATGGCCAGGCAATAAAGGAC-3′
Ethylene-responsive transcription Factor 1 (EU543659)	***ERF1***	F:5′-TGCTGCTTTTCATTTTCGTG-3′ R: 5′-AGGCGCTGTAAGAGGCATAG-3′

### Statistical analysis

The results were analysed statistically using one-way analysis of variance (ANOVA) followed by Duncan’s test and significant differences were identified (*p* < 0.05) using XLSTAT software (version 2014.5.03). The data are presented as the means of 3 replicates ± standard errors (SE). The Pearson correlation coefficient and principle component analysis (PCA) between different parameters and different SNP treatments were performed using XLSTAT software.

## Results

### Biosynthesis and characterization of sulfur nanoparticles (SNPs)

**Fig. 2:**
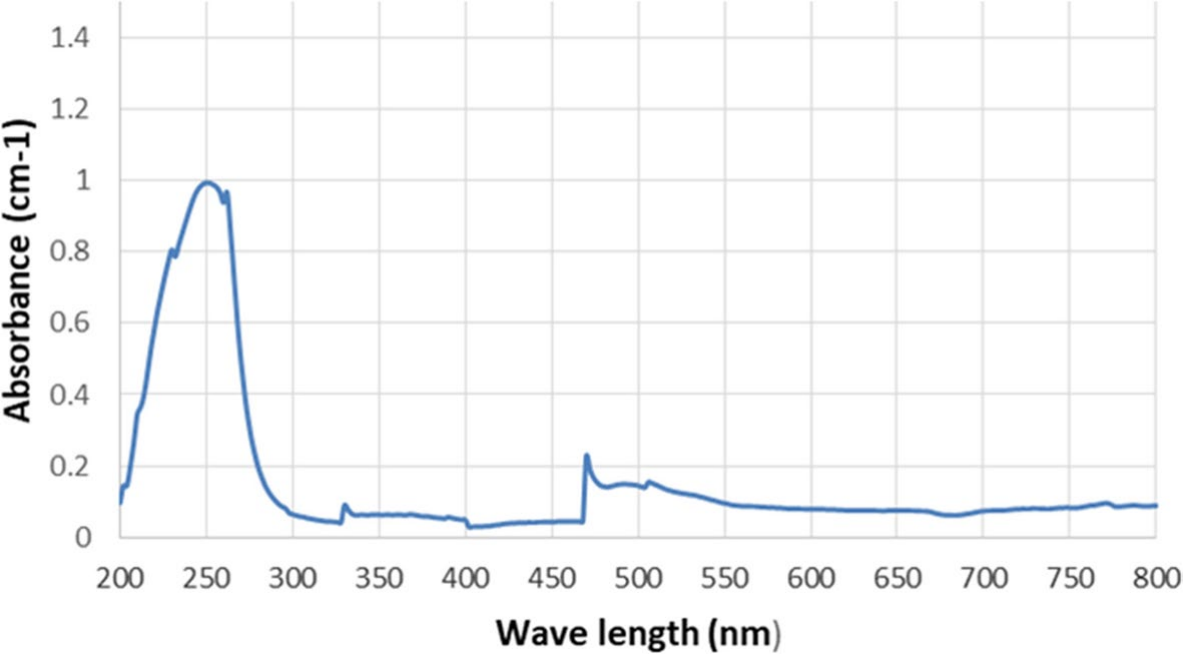
UV–vis spectra of green synthesized SNPs

The biogenic conversion of S ions into S nanoparticles was visualized through the change in the color of the reaction mixture from brown to yellowish-white over time (Fig. 1). This was confirmed by measuring the UV–visible spectra of the solution, which revealed a surface plasmon resonance (SPR) absorption band. The UV–visible spectrum of SNPs within the range of 200–800 nm revealed highly single-band absorption with a maximum peak at 250 nm, indicating SNPs (Fig. 2).

**Fig. 3:**
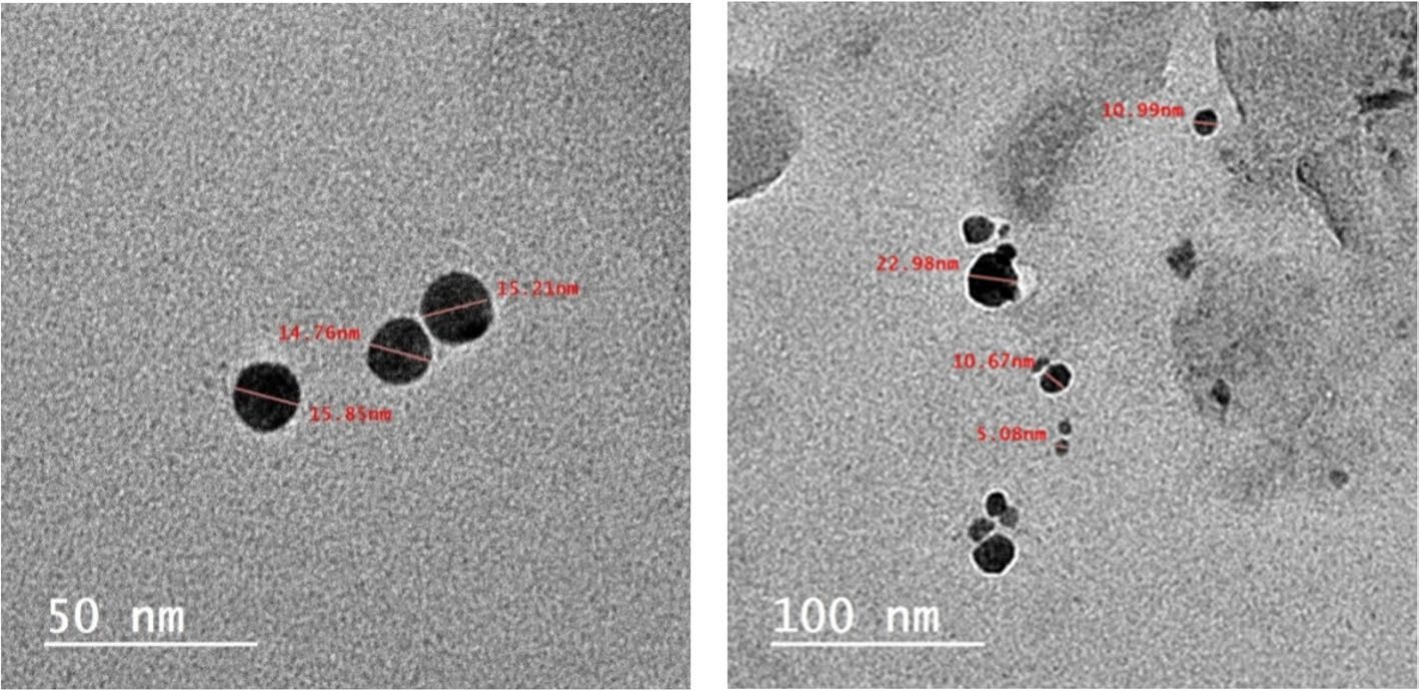
TEM images observed for sulfur nanoparticles at 50, and 100 nm magnification

In addition, TEM was performed to determine the size and morphology of the produced SNPs. The TEM images show that the particles exhibit a small size disparity. A spherical shape of the SNPs was observed, and their size ranged from 3.75 to 40.69 nm, with a mean of 10.98 ± 2.91 nm (Fig. 3).

**Fig. 4:**
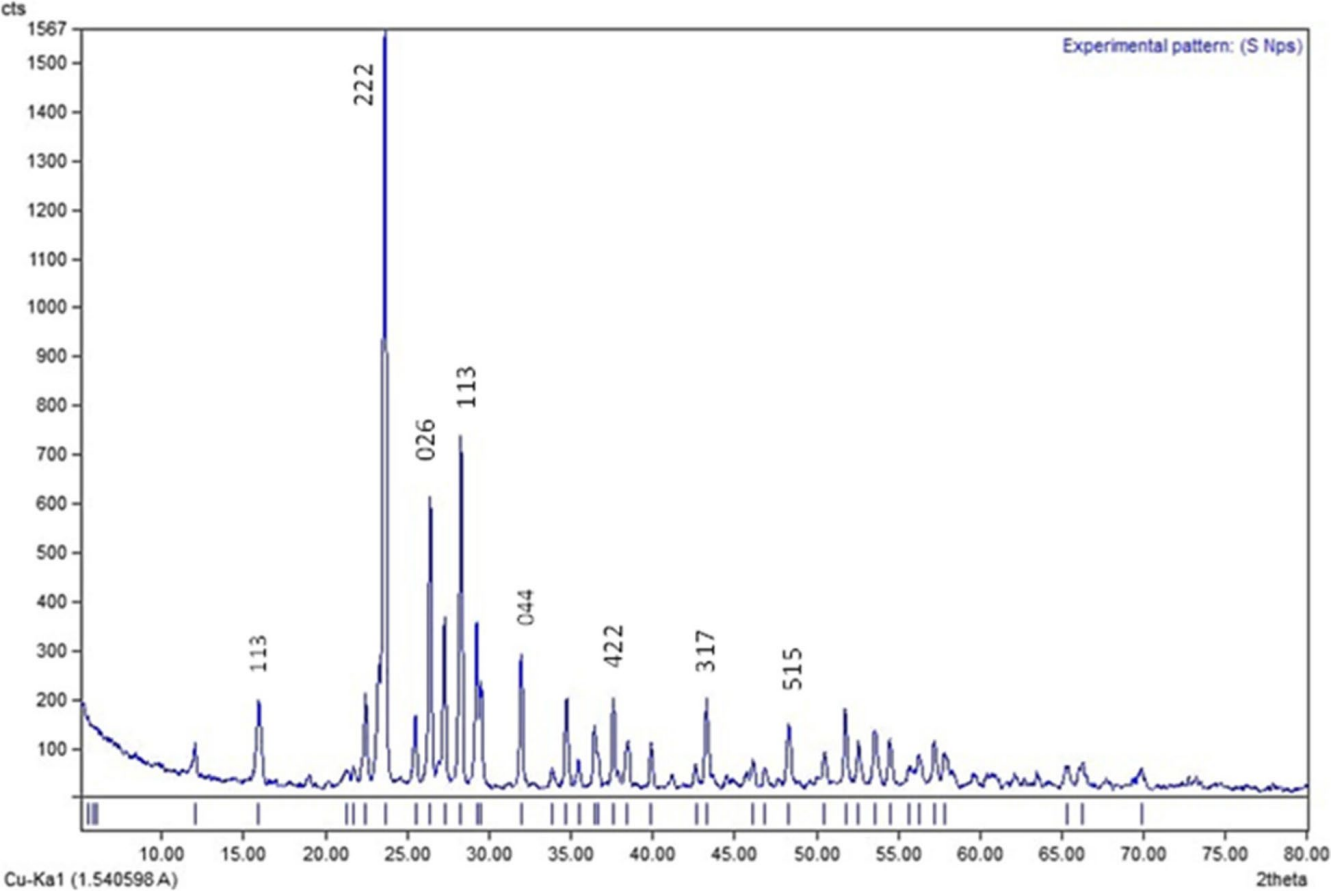
Characterization of green synthesized SNPs by XRD

Furthermore, the XRD pattern presented in Fig. 4 shows several sharp peaks, indicating the polycrystalline nature of the green synthesized SNPs. The 2θ peaks at 15.23°, 23.18°, 26.39°, 27.57°, 31.26°, 37.59°, 42.52°, and 47.52° are attributed to the sulfur crystals at (113), (222), (040), (313), (044), (422), (319), and (515), respectively. The SNPs are crystalline, and the relative position and intensities of the diffraction peaks aligned with those of standard sulfur (International Centre for Diffraction Data (ICDD), No. 00–008-0247).

**Fig. 5:**
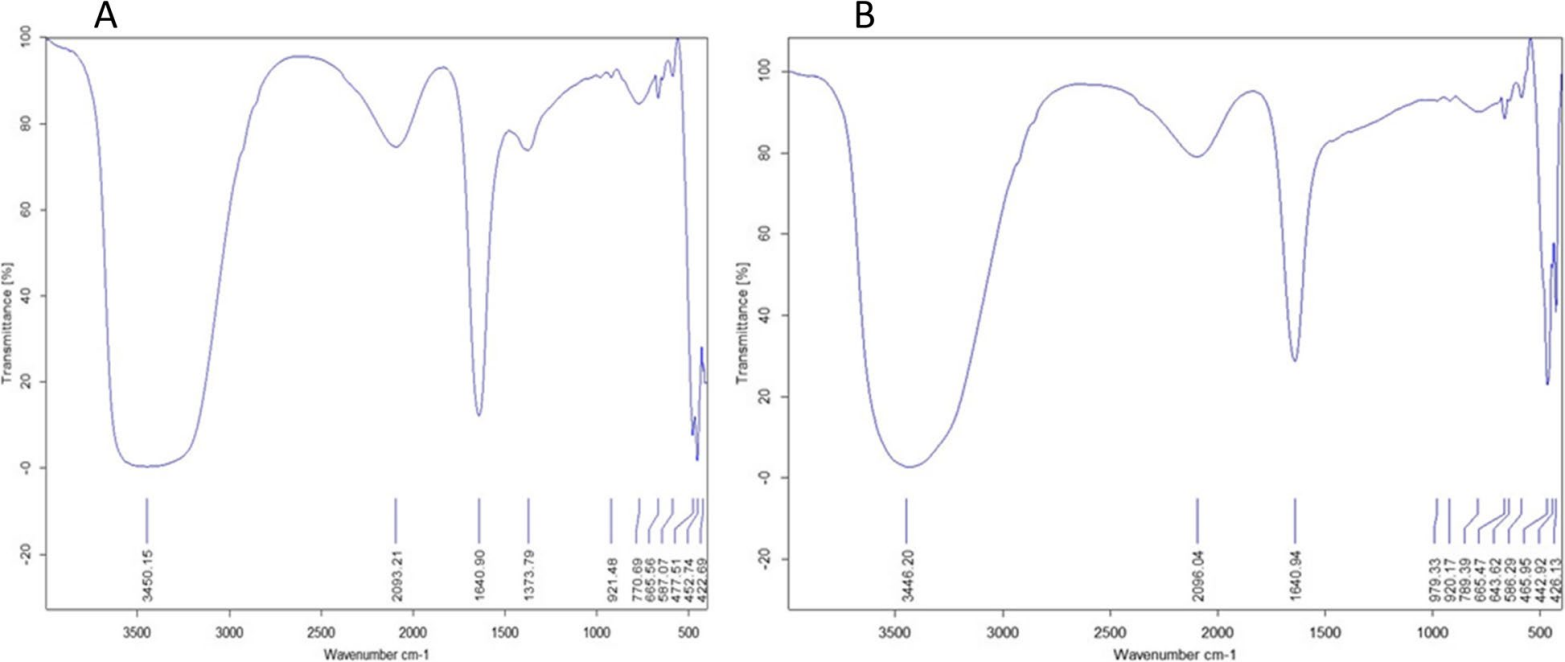
FTIR spectrum of **A**)* M. oleifera* leaves extract and **B**) green synthesized SNPs

Moreover, FT-IR analysis was performed to determine the potential biomolecules involved in the capping and stabilization of the biosynthesized SNPs. The transmittance band ranging from 400 cm^−1^ to 4000 cm^−1^ was recorded for the FTIR spectra as depicted in Fig. 5 A. The obtained FTIR spectrum of the *M. oleifera* leaf extract (Fig. 1d) clearly revealed several absorption peaks at 3450, 2093, 1640, 1373, 921, 770, 665, 587, 477, 452, and 422 cm^−1^. Similarly, the prominent peaks observed in the FTIR spectrum of *M. oleifera* leaves were also present in the FTIR spectrum of SNPs, as shown in Fig. 5B. However, there was a minor shift in the positions of these peaks at 3446, 2096, 789, 586, 465, 442, and 426 cm-1.

### Growth parameters and photosynthetic performance (Fv/fm)

**Fig. 6:**
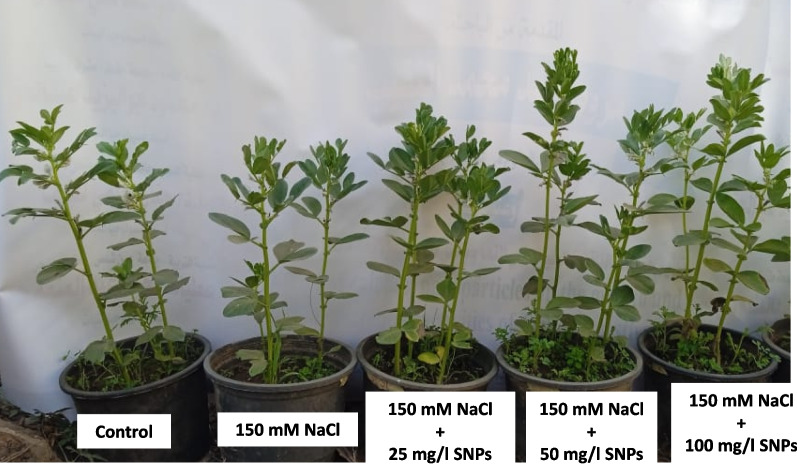
Effect of different concentrations of SNPs (0, 25, 50 and 100 mg/l) on faba bean growth under salt stress (150 mM NaCl)

In the present study, *V. faba* L. plants that were exposed to a concentration of 150 mM NaCl exhibited noticeable signs of stress, which manifested as a decrease in various growth parameters, as indicated in Table 2. Compared with the control plants, the salt-stressed plants exhibited significant decreases in shoot and root length (26.6 ± 2.02, 13.9 ± 2.07 cm), and lower fresh and dry weights (16.5 ± 1.06, 1.8 ± 0.25 gm), (38 ± 1.15, 29 ± 1.7, 21.9 ± 0.9, 2.4 ± 0.3, respectively). Plants sprayed with different concentrations of SNPs (25, 50, and 100 mg/l) showed improvements in growth criteria (Fig. 6). The most valuable dose was 50 mg/l, which markedly increased shoot length (30.7%), root length (42.6%), fresh weight (16.7%), and dry weight (62.5%) compared to those of the corresponding control. In addition, under normal growth conditions (0.79 ± 0.004), the Fv/Fm of leaves under salt stress (150 mM NaCl) decreased (0.73 ± 0.01). Compared with salt treatment, treatment with 50 mg/l SNPs significantly improved the Fv/Fm value by 8.3% (Table 2).

**Table 2:** Effects of different concentrations of SNP (0, 25, 50 and 100 mg/l) on the growth parameters and photosynthetic performance of faba bean plants under salt stress

Treatments	Shoot length (cm)	Root length (cm)	Fresh wt (g)	Dry wt (g)	Fv/Fm
Control	38^**bc**^ ± 1.15	29^**c**^ ± 1.7	21.9^**b**^ ± 0.9	2.4^**b**^ ± 0.3	0.79^**ab**^ ± 0.01
150 mM NaCl	26.5^**d**^ ± 2.02	13.9^**d**^ ± 2.1	16.5^**c**^ ± 1.1	1.8^**c**^ ± 0.2	0.74^**bc**^ ± 0.008
150mM NaCl + 25mg/l SNPs	33.56^**cd**^ ± 1.9	33.56^**bc**^ ± 1.9	23.26^**ab**^ ± 1.2	2.46^**bc**^ ± 0.1	0.76^**b**^ ± 0.011
150mM NaCl + 50mg/l SNPs	49.66^**a**^ ± 4.4	41.36^**a**^ ± 1.2	25.56^**a**^ ± 0.8	3.9^**a**^ ± 0.1	0.81^**a**^ ± 0.001
150mM NaCl + 100mg/l SNPs	46.66^ab^ ± 4.6	37.5^**ab**^ ± 1.8	24.76^**ab**^ ± 0.7	3.03^**ab**^ ± 0.3	0.79^**ab**^ ± 0.009

### Changes in osmolyte contents

**Fig. 7:**
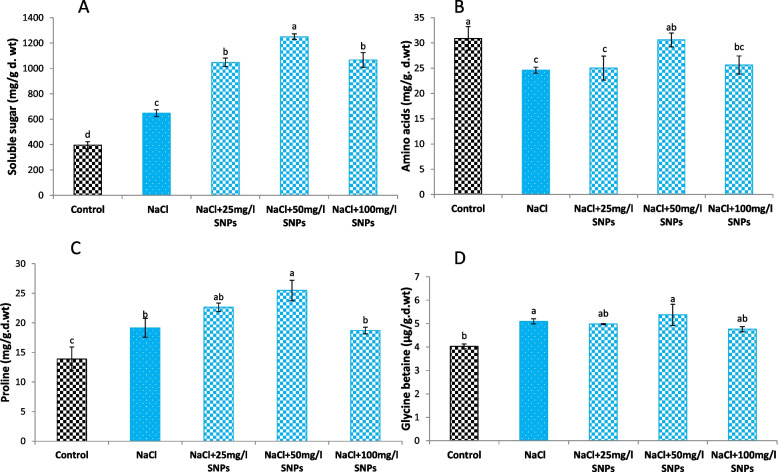
Levels of (**A**) soluble sugars, (**B**) amino acids, (**C**) proline, and (**D**) glycine betaine in the leaves of faba bean plants treated with SNPs under salt stress. Different letters denote significant variations at 0.05 level

Compared with the control, the 150 mM NaCl significantly elevated the total soluble sugar content (Fig. 7A). In addition, compared with both the salt dose and control treatments, all SNP treatments markedly increased the soluble sugar content. Compared with the control treatment, treatment with 50 mg/l SNPs was the most effective and increased the soluble sugar content by 215.6%. In addition, relative to the control, the salinity dose (150 mM NaCl) significantly decreased the amino acid content. Nevertheless, SNPs treatments exhibited pronounced increases in amino acid values in comparison to control. The highest amino acid content (30.57 mg/g d.wt) was recorded in the 50 mg/l SNPs-treated plants. Furthermore, the salinity stress dose significantly increased proline and GB contents by 36.6% and 26.5%, respectively, over those of the control (Fig. 7C, D). Similarly, compared with those in the control treatment, the proline and GB contents in the SNPs treatment group increased by 83.15% and 33.5%, respectively, and their maximum increases were recorded at 50 mg/l.

### Oxidative stress biomarkers

**Fig. 8:**
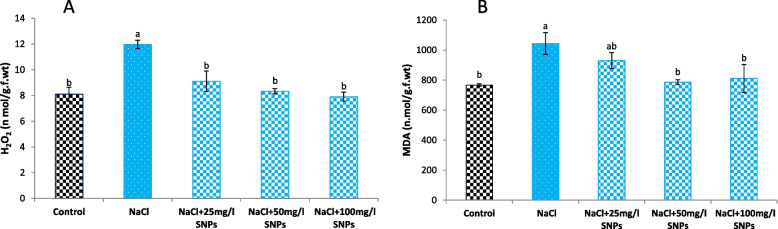
Levels of stress biomarkers ((**A**) H_2_O_2_, and (**B**) MDA) in the leaves of faba bean plants treated with SNPs under salt stress. Different letters denote significant variations at 0.05 levels

As widespread stress intensity indicators in plants, stress marker (MDA and H_2_O_2_) contents were determined to investigate whether the application of SNPs alleviated salinity oxidative stress in faba bean plants. The results showed that the salinity dose (150 mM NaCl) led to significant increases in the MDA and H_2_O_2_ concentrations compared to control plants (36.06 and 47.8%, respectively) (Fig. 8). Compared with salinity stress, SNP treatment markedly decreased MDA and H_2_O_2_ levels.

### Nonenzymatic and enzymatic antioxidants in faba bean Leaves

**Fig. 9:**
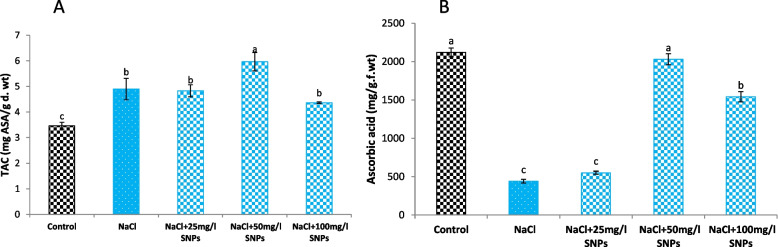
Levels of (**A**) total antioxidant capacity (TAC) and (**B**) ascorbic acid in the leaves of faba bean plants treated with SNPs under salt stress. Different letters denote significant variations at 0.05 levels

The results showed that the salinity dose (150 mM NaCl) significantly elevated the total antioxidant capacity (TAC) by 41.45% compared to the control. Similarly, SNPs application markedly enhanced the TAC, especially at 50 mg/l SNPs, which exhibited the highest TAC 72.11% greater than that of the control (Fig. 9A).

The ascorbic acid (ASA) content of faba bean leaves significantly declined as a result of salt stress in all treatments except for the 50 mg/l SNPs which restored the ASA content to the control level (Fig. 9B).

**Fig. 10:**
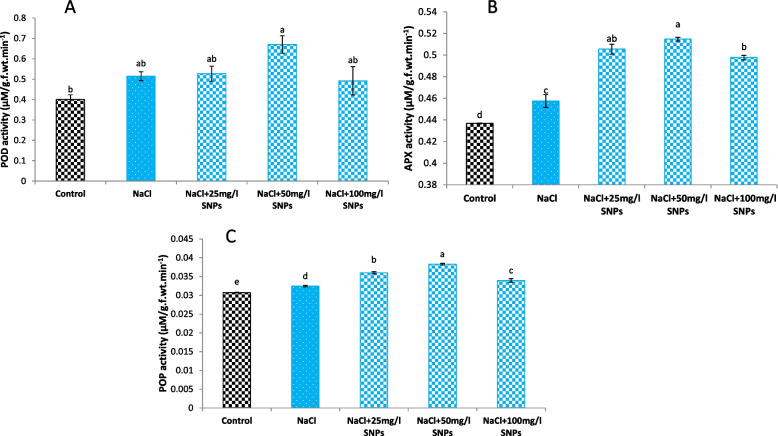
Levels of (**A**) APX, (**B**) POD, and (**C**) PPO in the leaves of faba bean plants treated with SNPs under salt stress. Different letters denote significant variations at 0.05 level

Regarding the antioxidant enzymes, the salinity dose enhanced the activity of APX, POD, and PPO by 4.7%, 28.18%, and 5.59%, respectively, compared to control. The foliar application of SNPs escalated the enzyme activities more than both the control and salinity dose treatments. Compared with the control, treatment Treatment with 50 mg/l SNPs resulted in the greatest increase in APX, POD, and PPO activity by 17.8%, 76.01%, and 24.6%, respectively (Fig. 10).

### Expression profiles of selected genes at different SNPs treatments on faba bean plants

**Fig. 11:**
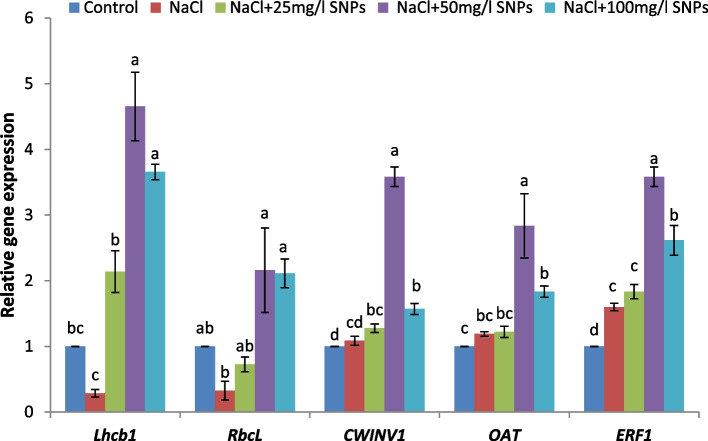
Expression levels of stress-responsive genes (*Lhcb1, RbcL, CWINV1, OAT,* and* ERF1*) in the leaves of faba bean plants treated with SNPs under salt stress

The expression patterns of five stress-responsive genes involved in plant development were examined (Fig. 11). The results showed that compared with the control, the 150 mM NaCl treatment downregulated the gene expression of *Lhcb1, RbcL,* and *CWINV1*, but significantly upregulated the expression of the *ERF1* gene*.* In contrast, the application of SNPs increased the expression levels of *Lhcb1, RbcL, CWINV1*, *OAT,* and* ERF1* genes compared with those in the control. The greatest changes in the expression of these genes were detected in plants sprayed with 50 mg/l SNPs, whose expression increased 3.6-, 1.6-, 3.7-, 1.8-, and 2.5-fold, respectively.

### Pearson correlation coefficient

**Fig. 12:**
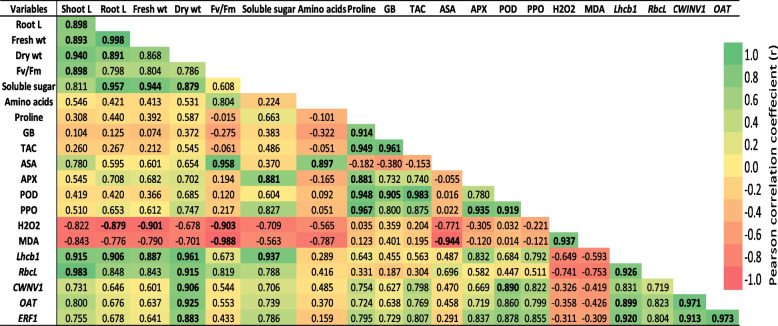
Pearson correlation of growth and physiological parameters, and expression levels of stress-responsive genes measured at different SNPs concentrations in faba bean plants under salt stress

Pearson’s simple correlation was applied to discern the relationships between the physiological and molecular attributes recorded in different treatments with SNPs application (Fig. 12). The results indicated that the proline and glyceine betain contents and total antioxidant capacity (TAC) were strongly correlated with each other, and were positively correlated with antioxidant enzymes (APX, POD, and OPP). In addition, the growth parameters were positively correlated with *Lhcb1* and *RbcL* expression levels. Furthermore, the expression level of *ERF1* gene was significantly correlated with *CWINV1* and *OAT* expression levels. On the other hand, MDA and H_2_O_2_ contents were negatively correlated with the expression levels of *Lhcb1, RbcL, CWINV1,* and *OAT* genes.

## Discussion

Salinity is a global conundrum that deleteriously affects various agricultural crops, hinders plant growth, and reduces crop yield [46]. Many studies have demonstrated that the external application of sulfur, as a mineral additive can overcome plant stresses like salinity [47]. Nanotechnology has gained special importance regarding salinity stress resilience in crop plants. Green synthesis involves synthesizing nanomaterials or nanoparticles using biological sources, with a focal point on the development of more convenient techniques for the production of eco-friendly, nontoxic, benign nanoparticle [48]. Hence, from this perspective, the present study assessed the efficacy of *M. oleifera* leaf extract in the green biosynthesis of sulfur nanoparticles, which in turn were applied to enhance salinity resilience in the faba bean plant. The biogenic conversion of S ions into S nanoparticles was visualized through the change in the color of the reaction mixture from brown to yellowish-white over time (Fig. 1). The observed color change was attributed to the excitation of SPR by the SNPs [23, 49]. The resultant UV–visible spectrum showed highly symmetrical single-band absorption with a peak at 250 nm, signifying SNPs. Previous studies have shown that SNPs exhibit absorption spectra within the range of 245–300 nm [49, 50]. Additionally, the XRD analysis demonstrated diffraction peaks that aligned with the standard sulfur and indicated the polycrystalline structure of the synthesized SNPs.

In the IR spectrum of *M. oleifera* leaf extract, the strong absorption peak at 3450.18 cm-1 can be attributed to the hydroxyl (-OH) groups of stretching H-bonded alcohols and phenols. In the synthesized SNPs, this band was shifted, which is probably due to the attachment of sulfur ions to H atoms [49]. The band at position 2093 may be attributed to the carbonyl group (C = O) in carboxylic acids, ketones, and aldehydes [51]. The band observed at 1640 cm^−1^ in both the leaf extract and the synthesized SNPs is characteristic of the N–H functional group that is commonly found in the protein. The absorption bands at 770, 665, and 587 to 422 cm^−1^ for the *M. oleifera* leaf extract and green synthesized SNPs, respectively, could be due to the aromatic C-H and C–C skeleton-bending monosubstituted aromatics. Overall, FT-IR analysis of both the *M. oleifera* leaf extract and the biosynthesized SNPs revealed characteristic peaks corresponding to functional groups such as -OH, -N–H, and C = O. Consequently, it can be suggested that the reduction process leading to the formation of SNPs and their stabilization is performed by water-soluble aromatic compounds like phenolics and flavonoids, in addition to proteins. Similar findings have been reported for silver NPs via Gupta et al. [52] and for green synthesized SNPs via Paralikar & Rai [49].

Plant growth features are useful indicators for assessing the impacts of salinity on plants [6]. In the current study, our results revealed that foliar treatments of faba bean with different concentrations of SNPs were notably effective in mitigation the adverse effects of salinity-induced growth reduction, whereas the level of mitigating was found to be dependent on the dose of SNPs applied. Compared with the salinity dose, the most effective SNP dose was 50 mg/l (Table 2). Similar to our findings, Salem et al. [23] found that SNPs treatment augmented tomato growth, and Najafi et al. [53] reported similar results for *Cinnamomum zeylanicum* plants. The beneficial effects of SNPs can be attributed to their sulfur is necessary in the formation of several biologically active compounds involving cysteine, 5-adenylyl sulfate, glutathione, coenzyme A, and chlorophyll [54, 55]. Therefore, sulphur plays a critical role in enhancing cellular function by restoring osmolyte balance, which ultimately promotes salt resistance in plant crops [56, 57], which could explain the ability of SNPs to augment the growth of salinity-stressed faba bean plants.

Moreover, significant decreases in chlorophyll fluorescence were recorded in salt-stressed faba bean plants compared to those under normal growth conditions. Additionally, Kafi [58] further highlighted that salinity stress had a negative effect on the light-utilization efficiency, decreasing of the Fv/Fm of broad bean plants, and disturbance of the electron flow from photosystem II (PSII) to electron acceptors hindered the regeneration of CO2 acceptors. The Results revealed that the use of SNPs significantly increased chlorophyll fluorescence (Fv/Fm) in salt-stressed faba bean plants. According to Salem et al. [23], the beneficial impact of SNPs on photosynthetic pigments is due to their interaction with other organic molecules, leading to the formation of organo-sulfur compounds and stimulating chlorophyll biosynthesis in leaves. Najafi et al. [53] reported that the photosynthetic pigment content was enhanced under SNP application, which can improve the photosynthesis rate and thereby augment the growth and biomass production of plants.

Under salt stress, ROS accumulate in plant cells, resulting in severe oxidative damage. To alleviate the harmful impacts of salt stress, plants stimulate their self-defense systems through the upregulation of osmolytes such as soluble sugars, amino acids, proline and glycine betaine [59]. Osmolytes help maintain membrane integrity and fluidity, thereby preserving cellular functions and minimizing electrolyte leakage under stress [60]. Our results revealed that a salt stress dose of 150 mM NaCl enhanced the soluble sugar, proline and glycine betaine concentrations while reducing the amino acid content in faba bean plants. This decline in amino acids is intricately linked to the diminished activity of the nitrate reductase enzyme (NR) under soil salinization, a consequence of decreased nitrate (NO3˗) uptake and heightened chloride (Cl˗) uptake by plants in response to salt stress, as elucidated by. The suppression of NR activity disrupts the protein synthesis mechanism, exerting a negative impact on plant growth and development [61]. However, in the present study, SNPs application efficiently restored the normal levels of osmolytes in faba bean plants, especially at a concentration of 50 mg/l. This amino acid enhancement could be attributed to the enhancement of nitrogen metabolism by SNPs, which may be attributed to their ability to balance nitrogen uptake, repaire the carbon skeleton, protect nitrogen assimilating enzymes, and upregulate sulfur [56]. Mogazy and Hanafy [62] reported greater levels of osmolytes in salt-stressed faba bean plants treated with foliar ZnO NPs when than in stressed plants. Furthermore, proline can accumulate in plant cells to maintain the cellular osmotic potential and prevent water loss [63].

For indicators of oxidative stress, the MDA and H_2_O_2_ contents were determined in the leaves of the faba bean plants. In this study, the H_2_O_2_ and MDA contents significantly increased with salt stress in the faba bean (36.06%), compared to the control. Accordingly, it appears that salinity stress can disrupt various cellular components, such as lipids and protein molecules, in addition to disrupting the structural balance of the cell membrane. However, in the present study, the uses of SNPs significantly overcome the adverse effects of salt stress by decreasing the MDA and H_2_O_2_ contents. This decrease may be due to SNPs mediating cell membrane recovery, which improves plant vigor and reduces the risk of injury caused by salinity stress. In line with our results, Najafi et al. [53] found that applying green synthesized SNPs to *Lactuca sativa* led to a decline in H_2_O_2_ and MDA contents, reduced free radicals, and improved plant growth conditions.

Numerous studies have shown that salinity stress triggers the production of ROS in plants, which causes the levels of antioxidant enzymes and nonenzymatic antioxidants to increase as a defense mechanism, which serves as an indication of ROS production and signifies the plant's ability to adapt to ROS [7, 64]. In the present work, the results demonstrate that cultivating faba bean under saline conditions leads to an elevation in TAC and the activity of antioxidant enzymes (APX, POD, and PPO) while decline ascorbic acid. This decline in ascorbic acid is intricately related to salt stress, which can inhibit the biosynthesis of ascorbic acid in plants by disrupting the activity of enzymes involved in its synthesis pathway [65]. In addition, foliar treatments with SNPs, especially at 50 mg/l, significantly enhanced TAC, ascorbic acid and antioxidant enzyme activities compared to salt-stressed plants. The stimulation of APX and POD activities is responsible for detoxifying H_2_O_2_, thus preventing any harm it may cause [66]. In addition, the increased activity of PPO during stress conditions indicates its capacity to oxidize and degrade toxic substances, which are generally reported to accumulate during salt stress [67, 68]. Nanomaterials can ameliorate the antioxidant defense system (enzymatic and nonenzymatic compounds) and thus neutralize the overproduction of ROS, via silver in marigold silver in marigold [69], copper in ashwagandha [70] and selenium in lemon balm [71]. The augmentation of enzymatic and nonenzymatic activities enhanced salt stress resilience, played a vital role in sustaining the proper development of the faba bean plant, and potentially explained the improvements observed in various growth parameters.

Photosynthesis, which serves as the foundation for plant growth, is a highly sensitive physiological process under plant stress [72]. Light energy is absorbed by photosynthetic antenna proteins. These proteins are further categorized into LHCA and LHCB, which assist PSI and PSII, respectively. PSII is a critical component involved in the photoreaction of photosynthesis, and Lhcb proteins also play a role in modulating plant responses to abiotic stress conditions [73, 74]. Our results revealed that salt stress downregulated the *Lhcb1* gene in faba bean plants. Salt stress decreases the effectiveness of light energy and the electron transport process in PSII. Conversely, foliar application of SNPs (50 mg/l) upregulated *Lhcb1* gene expression in salt-stressed plants. This result is consistent with that of Zarasvand [75], who reported upregulated expression levels of *Lhcb* genes as a result of ZnO NPs treatment in *Arabidopsis thaliana* plants. Additionally, the results of the Pearson correlation showed that there was a significant association between the expression levels of *Lhcb1* gene and growth parameters, suggesting that the activity of this gene can play a role in regulating plant development and biomass accumulation. (Fig. 12). This finding is in line with Xia et al. [76], who found that a significant correlation between *Lhcb1* and important agronomic traits like plant height and leaf color in barley.

Ribulose-1,5-bisphosphate carboxylase/oxygenase (Rubisco) is a pivotal enzyme in photosynthetic carbon assimilation, that consists of two types of protein subunits: the large chain and the small chain [77, 78]. The results revealed that salinity stress negatively affected on the expression level of the *rbcL* gene in faba bean leaves. This can be attributed to a decrease in the uptake of essential Mg^2+^ ions, which are necessary for Rubisco function. This leads to a reduction in chlorophyll biosynthesis and inhibition of the electron transport system [79]. Additionally, it has been proposed that salt stress indirectly inhibits the *rbcL* gene by promoting ROS, which disrupts the structure of chloroplast thylakoid membranes [80]. The current findings suggest a positive effect of SNPs on *rbcL* gene expression in salinity-stressed plants. This positive effect may be attributed to increased chlorophyll content, improved PSII efficiency, maintenance of chloroplast membrane integrity, and reduced sodium ion (Na^+^) uptake [80].

Both the ornithine-aminotransferase (*OAT*) gene and the cell wall invertase gene (*CWINV1*) play critical roles in mediating the plant response to salt stress. While OAT contributes to osmotic adjustment and oxidative stress tolerance through proline biosynthesis, CWINV1 regulates carbohydrate metabolism and resource allocation to support plant growth and stress adaptation [81, 82]. The coordinated expression of these genes is essential for enhancing stress tolerance and maintaining cellular homeostasis in saline environments [83]. Treatment with 50 mg/l SNPs significantly upregulated the expression levels of soluble sugar and proline-related genes, *CWINV1* and *OAT*, respectively, compared to salt-stressed plants. The upregulation of *CWINV1* gene expression represents a strategic response to maintain cellular homeostasis and ensure the efficient utilization of carbon resources under stress conditions [84]. A direct correlation was observed between OAT activity and the accumulation of proline in radish cotyledons and in rice leaves subjected to NaCl treatment [85, 86]. Roosens et al. [87] confirmed a correlation between salt tolerance and OAT activity in *Arabidopsis* plants.

*Ethylene-responsive transcription factor 1* (*ERF1*) belongs to the AP2/ERF transcription factor family found in plants [88]. ERF TFs play a significant role in the response to abiotic stress [89], the transduction of hormonal signals [90], and regulating the development of the abscission zone [91]. Our results showed that salt stress enhanced the expression level of *ERF1* gene in faba bean. These findings agree with Oliveira et al. [92], who found that overexpression of *ERF1* in tobacco plants led increased tolerance to both salt and drought stresses. Pearson correlation analysis revealed that *ERF1* was significantly correlated with *OAT* and *CWINV1* (Fig. 12). This finding is in line with Li et al. [93], who found that overexpression of *VaERF3* in *Vigna angularis* leads to an elevation in proline content and the development of salt resistance in Arabidopsis. In addition, Zhang et al. [94] found that *JERF1* upregulated the expression of osmolyte synthesis-related genes in rice.

## Conclusions

In conclusion, the findings of this study underscore the potential of SNPs as ecofriendly nanofertilizers for mitigating the adverse effects of salt stress on growth and physiology of faba bean plants. The observed improvements in growth parameters, osmolyte accumulation, antioxidant defense systems, and gene expression profiles highlight the multifaceted mechanisms underlying the stress-mitigating effects of SNPs. Overall, a dual-functional SNPs nanofertilizer that nourishes plant growth while enhancing salt stress resilience in faba bean offers promising opportunities for faba bean production in saline-affected regions. Therefore, future research should focus on optimizing the application methods and dosages of SNPs for different crop varieties and soil conditions to further enhance their efficacy in mitigating salinity stress.

## Data Availability

All data supporting the findings of this study are already included in this published manuscript.

## Abbreviations

SNPs: Sulfur nanoparticles
XRD: X-ray diffractometer
TEM: Transmission electron microscopy
FTIR: Fourier Transform Infrared Spectroscopy
APX: Ascorbate peroxidase
POD: Peroxidase
PPO: Polyphenol oxidase
*OAT*: Ornithine-aminotransferase
*ERF1*: Ethylene-responsive transcription factor 1
*RbcL*: Ribulose bisphosphate carboxylase large chain-like
*Lhcb1*: Chlorophyll a-b binding protein of LHCII type 1-like
*CWINV1*: Invertase I
Fv/fm: Photosynthetic performance
GB: Glycine betaine
TAC: Total antioxidant capacity
ASA: Ascorbic acid content
H_2_O_2_: Hydrogen peroxide
MDA: Malondialdehyde
PCA: Principle component analysis
ANOVA: Analysis of Variance
